# Formulation of a reactive oxygen producing calcium sulphate cement as an anti-bacterial hard tissue scaffold

**DOI:** 10.1038/s41598-021-84060-9

**Published:** 2021-02-24

**Authors:** Thomas J. Hall, Erik A. B. Hughes, Hamzah Sajjad, Sarah A. Kuehne, Melissa M. Grant, Liam M. Grover, Sophie C. Cox

**Affiliations:** 1 School of Chemical Engineering, University of Birmingham, Edgbaston, Birmingham, B15 2TT Northern Ireland UK; 2 NIHR Surgical Reconstruction and Microbiology Research Centre, Queen Elizabeth Hospital, Birmingham, B15 2TH Northern Ireland UK; 3 School of Dentistry, Institute of Clinical Science, University of Birmingham, Edgbaston, Birmingham, B5 7EG Northern Ireland UK; 4 Institute of Microbiology and Infection, University of Birmingham, Edgbaston, Birmingham, B5 7EG Northern Ireland UK

**Keywords:** Drug discovery, Health care, Medical research

## Abstract

Prophylactic antibiotic bone cements are extensively used in orthopaedics. However, the development of antimicrobial resistance to antibiotics, demonstrates a need to find alternative treatments. Herein, an antimicrobial honey (SurgihoneyRO-SHRO) has been successfully incorporated into a calcium sulphate (CS) based cement to produce a hard tissue scaffold with the ability to inhibit bacterial growth. Antimicrobial properties elicited from SHRO are predominantly owed to the water-initiated production of reactive oxygen species (ROS). As an alternative to initially loading CS cement with SHRO, in order to prevent premature activation, SHRO was added into the already developing cement matrix, locking available water into the CS crystal structure before SHRO addition. Promisingly, this methodology produced > 2.5 times (715.0 ± 147.3 μM/mL/g) more ROS over 24 h and exhibited a compressive strength (32.2 ± 5.8 MPa) comparable to trabecular bone after 3 weeks of immersion. In-vitro the SHRO loaded CS scaffolds were shown to inhibit growth of clinically relevant organisms, Staphylococcus aureus and Pseudomonas aeruginosa, with comparable potency to equivalent doses of gentamicin. Encouragingly, formulations did not inhibit wound healing or induce an inflammatory response from osteoblasts. Overall this study highlights the prophylactic potential of CS-SHRO cements as an alternative to traditional antibiotics.

## Introduction

Hard tissue defects can arise following disease or trauma, including the excision of infected tissue and benign or cancerous growths. While bone exhibits a capacity to “self-heal” small defects, those larger than critical size require surgical intervention. In this case, bone graft or biomaterials may be used to repair the defect and provide a scaffold for new hard tissue deposition. It is essential that these materials are well tolerated by the body (i.e. biocompatible), resorbed at a rate that matches bone formation, cost-effective, easily stored and easy to apply within the clinical setting. To this end, there has been a vast emergence of calcium-salt based cement systems in recent decades^1^.

In particular, calcium sulphate cements (CSC) have been widely adopted clinically. This success is due to the formation of an injectable paste that may be easily and directly delivered to the defect site. Advantageously, the setting reaction of calcium sulphate, which progresses through a dissolution–precipitation reaction, is compatible within the physiological environment (Eq. 1). The cement can therefore harden in situ to provide a scaffold for new bone formation. In situ setting gives rise to beneficial properties that can enhance patient recovery and well-being such as custom fitting of a defect and the loading of antibiotics.

1 $$ {\text{CaSO}}_{4} \cdot {0}{\text{.5H}}_{{2}} {\text{O}} + 1.5{\text{H}}_{2} {\text{O}} \to {\text{CaSO}}_{4} \cdot 2{\text{H}}_{2} {\text{O}} $$

Antibiotics are vital to modern medicine, helping to protect patients from infection that may be the result of injury, a compromised immune system, or the undertaking of procedures such as surgery^2,3^. It is therefore alarming that bacteria are rapidly evolving resistance to many antibiotics, which may be attributed to global misuse^4,5^. Gentamicin, a broad spectrum aminoglycoside antibiotic has been loaded into bone cements to provide a prophylactic effect since the early 1970’s^6–8^. Resistance to gentamicin, like all mainstream antibiotics, is ever increasing, a 2002 report by Thornes et al. stated that gentamicin is unsuitable for use in bone cements for revision surgery due to the development of resistance^9,10^. More recently it was determined by Miller et al.^11^ that in 88% of joint infections where gentamicin was used as a prophylactic, resistance to gentamicin was discovered^11^. As such, the development of novel antimicrobials that provide a viable alternative to commonly used prophylactics, as well as systems by which to enable efficacious delivery, are a healthcare priority^9,12^.

Reactive oxygen species (ROS) such as hydrogen peroxide, superoxide and hydroxyl ions are known to have antimicrobial properties with ROS reacting with thiol groups found in enzymes, proteins and DNA, inhibiting function^13,14^. Additionally, ROS can also oxidise bacterial cell walls and membranes preventing transport and disrupting electron transport, leading to cellular death^15–18^. These antimicrobial effects however are non-specific and therefore at high levels (> 100 µM) ROS can have deleterious effects on host cells^19^. Hydrogen peroxide (H_2_O_2_) has previously had limited use in wound care to treat and prevent infection, but due to its fast reactivity often cytotoxic doses (≥ 3%) are used. This is in order to keep the ROS concentration above the microbicidal level for a therapeutically relevant period of time^20–22^. A review by Zhu. et al.^23^, concluded that it would be beneficial to deliver lower, sustained doses of ROS. Extracellular concentrations of 25–50 µM have been found to be sufficient to induce bacteriostasis and concentrations of up to 500 µM will induce bacterial cell death^24,25^. Further to the direct antimicrobial action of ROS these molecules play an important role in the human body’s response to infection and wound healing^26^. This immune response occurs at ROS concentrations between 5 and 250 µM and drives the recruitment of endogenous macrophages which generate ROS concentrations of between 10 and 1000 µM^19^. Furthermore, ROS has also been found to support wound healing when concentrations are between 0.1 and 10 µM, specifically the processes of proliferation, differentiation, migration, angiogenesis and act as an anti-inflammatory^19,27,28^.

The production of ROS for its bactericidal capabilities is a mechanism widely utilised in nature. For example, this defence system can also be found in honey with ROS produced by the enzymatic oxidation of glucose (Eq. 2)^29^. Honey has been used for millennia in wound care applications and is mainly composed of three components, fructose (≈38%), glucose (≈31%) and water (≈17%)^30,31^. The remaining constituents are other sugars, acids, proteins, vitamins and minerals^32^. There are a number of medical grade products currently available, however due to compositional batch to batch variation, as may be expected with a natural product, its use in modern medicine is limited^33,34^. Furthermore, antimicrobial honey formulations that utilise production of ROS as the mode of action, are restricted due to the water sensitive nature of the reaction (Eq. 2). The addition of non-bound water prior to application would therefore reduce the shelf life and efficacy of the product.

2 $${\text{C}}_{6} {\text{H}}_{{12}} {\text{O}}_{6}  + {\text{H}}_{2} {\text{O}} + {\text{O}}_{2} \xrightarrow{{GOx}}{\text{C}}_{6} {\text{H}}_{{12}} {\text{O}}_{7}  + {\text{H}}_{2} {\text{O}}_{2}$$

SurgihoneyRO (SHRO) can be bioengineered from natural honey with no restrictions on floral origin, in order to provide consistent ROS production. This ROS production provides SHRO with its main mode of antimicrobial action^35^. In vitro and in vivo studies including that by Hall et al. have shown this class IIb medical grade honey has the capacity to treat infecting bacterial cultures, including both Gram-positive and Gram-negative bacteria^14,36–41^. This includes *Staphylococcus aureus,* one of the most frequently isolated pathogens in joint replacement surgery, as well as *Pseudomonas aeruginosa,* a World Health Organisation research and development priority 1 pathogen^7,42^. Drug resistant strains such as methicillin-resistant *Staphylococcus aureus* (MRSA) and vancomycin-resistant *Enterococcus faecium* were also eradicated^41^*.* Moreover, SHRO has been demonstrated to be more or equally as efficacious as both Activon manuka honey and Medihoney manuka honey as well as the two most commonly used wound care antimicrobials, iodine and silver^38,40^. In addition, SHRO has been shown to be effective at preventing the seeding of biofilms as well as reducing biofilm mass^38,43^. The promising potency demonstrated by SHRO makes it an interesting candidate for further investigation into its ability to replace more traditional antibiotics for use in infection prevention or treatment. To date, little work had been done to assess the capability of this water sensitive active with existing biomaterial delivery systems.

For the first time, this study highlights the potential to formulate a calcium sulphate and SHRO cement producing an antimicrobial regenerative scaffold. Herein, we address the challenging water-sensitive nature that accompanies the use of ROS producing honey products by limiting its exposure to free water. This is achieved by locking excess water within the crystal structure of the hydrated cement. Furthermore, key physicochemical properties of this novel cement are analysed to determine usability, including compressive strength, setting time and injectability. Alongside antibacterial testing a scratch assay was conducted in order to assess wound healing potential in vitro.

## Materials and methods

### Preparation of cement

Cements were prepared with a liquid to powder (L:P) ratio of 0.2 mL/g. Anti-bacterial derivatives contained 0.1 g of SHRO per gram of cement powder. Briefly, calcium sulphate hemihydrate (CaSO_4_·0.5H_2_O) powder (Sigma Aldrich, UK) was combined with distilled water and mixed for 30 s to form a workable paste before hardening (CS_Control). SHRO (Matoke Holdings, UK) was combined either as an initial constituent of the cement (CS_SHRO1) or combined with cement paste 4 min after the initial 30 s mixing time (CS_SHRO2).

Once mixed, pastes were cast into a split mold and placed upon a vibrating plate to reduce air bubbles within the final cement samples. The cement filled split mold was incubated at 37 °C for 12 h to produce 6 by 13 mm (D:H) cylindrical test specimens. Initial and final setting times were determined using Gillmore needle apparatus and following ASTM standard C266-15. Briefly, approximately 25 cm^3^ of cement paste was prepared and manipulated into a flat-topped pat suitable for multiple penetrations with Gilmore needles. The initial setting time was determined by vertically applying a 113.4 ± 0.5 g weighted needle with a tip diameter of 2.12 ± 0.05 mm to the surface of the pat. The initial setting time was taken as the point at which no appreciable indent was observed post addition of the liquid component. The final setting time was determined by vertically applying a 453.6 ± 0.5 g weighted needle with a tip diameter of 1.06 ± 0.05 mm to the surface of the pat. The final setting time was taken as the point at which no appreciable indent was observed post addition of the liquid component. Measurements were recorded at 10 min intervals in triplicate.

### Cement characterisation

Cement degradation was examined by static ageing. Individual cast cement cylinders were placed in a cylindrical container with a diameter of 22.1 mm and fully submerged in 2.5 mL of distilled water. At days 7, 14 and 21, assigned cement specimens were removed from ageing supernatant and dried at 60 °C prior to recording changes in mass in triplicate.

Mechanical testing was performed using a Zwick/Roell Z030 Universal testing rig equipped with a 50 kN load cell. Compression tests were undertaken at a loading rate of 2 mm/min. Following a specimen pre-load of 5 N, force versus deformation curves were recorded in triplicate. Tested samples were recovered for microstructural analysis of fracture surfaces.

Scanning electron microscopy (SEM) was used to image cement fracture surfaces. Imaging was performed using a Hitachi TM3030Plus tabletop instrument. Back scattered electron (BSE) and secondary electron (SE) were acquired with an acceleration voltage of 15 kV. All SEM samples were mounted onto aluminium stubs using double sided carbon discs before being gold coated in argon atmosphere to ensure conductivity using a K550X sputter coater (Quorum Technologies, UK) with the sputter current at 20 mA and coating time of 20 s.

Compositional changes were characterised by powder X-ray diffraction (XRD). Samples for XRD were prepared by powdering cement with a pestle and mortar prior to analysis. A Bruker D8 Advance instrument fitted with a Cu X-ray source (1.5418 Å) and LYNXEYE (1D mode) detector was used to obtain diffraction patterns between 10 and 50° 2θ. Step sizes of 0.02° were used with a step time of 0.3 s. Phase identification was performed with DIFFRAC.SUITE software (Bruker, USA). Acquired patterns were matched with those within the International Centre for Diffraction Data (ICDD) library. The software was also used to determine the respective compositions of resulting cement formulations.

A Renishaw inVia™ Raman microscope equipped with 633 nm laser and 1200 l/mm grating was also used to analyse samples. 3 accumulative scans were used to calculate an average. Scans were obtained at 1% power and 30 s exposure time. The baseline was then subtracted and cosmic rays were removed using WiRE™ software.

### Anti-bacterial properties of cements

Initially the capacity of the cements to generate hydrogen peroxide (H_2_O_2_), a reactive oxygen species (ROS), was determined. Cast cement cylinders (“Preparation of cement” section) were placed in a cylindrical container with a diameter of 22.1 mm and fully submerged in 2.5 mL of distilled water. Supernatant was sampled at 1, 3, 6 and 24 h as well as at 4 and 7 days. H_2_O_2_ was detected using a fluorometric hydrogen peroxide assay kit (Sigma Aldrich, UK) with three cements tested at each time point. Due to the instability of the H_2_O_2_ molecule, collection of samples was staggered, such that supernatant from each time points was sampled and assayed simultaneously.

Anti-bacterial activity of cements was also determined by an agar diffusion method. Nutrient agar plates were prepared by pouring 25 mL of sterilized nutrient agar into Petri dishes. Once solidified, condensation was eliminated by placing the agar filled Petri dishes in a 60 °C dry heat oven. Overnight cultures of *S. aureus.* (ATCC 29213) and *P. aeruginosa *(NCTC 13437) were prepared by inoculating one colony of each strain into 5 mL of Luria–Bertani (LB) broth (Sigma Aldrich, UK) and incubated at 37 °C for 16 h in a shaking incubator (150 rpm). Each overnight culture was then diluted to an optical density (OD_600_) value of 0.02, using an Evolution 300 UV–VIS spectrophotometer (Thermo Scientific, UK). The plates were then inoculated with bacteria, using a hockey stick spreader to ensure maximum coverage. A sterile hole borer (Sigma Aldrich, UK) was then used to create a 10 mm diameter well in the center of the inoculated agar. For the pre-set samples cement supernatant was sampled after 24 h, of which 250 µL was transferred to inoculated agar wells (CS_SHRO2 Dry). Alternatively, 250 µL of cement paste was directly injected into the well (CS_SHRO2 Wet). A gentamicin dose comparison was prepared at a concentration of 0.0024 g/mL. This equates to the same calculated mass of hydrogen peroxide released after 24 h from CS_SHRO2 scaffolds. Plates were then incubated at 37 °C for 24 h. Zones of inhibition (ZOI) measurements were measured using a ruler^37^. Experiments were performed in triplicate.

### Osteoblast cell migration and inflammatory response

500,000 osteoblast like cells (SAOS-2—Passage number 28) were seeded into each well of a Corning™ 24 well plate (Sigma Aldrich, UK) and incubated in a HeraCell 150i CO_2_ incubator (Thermo Scientific, UK) at 37 °C with 5% CO_2_ for 24 h. Cement samples were then suspended in the cell media using Falcon™ trans-well inserts with a pore size of 0.4 μm (Fisher Scientific, UK).

Osteoblast migration was assessed by means of an in vitro scratch assay which saw a vertical scratch across the well made using a pipette tip. The cells were subsequently washed using phosphate buffered saline (PBS) (Thermofisher, UK) to remove debris and McCoy’s 5A modified media (Fisher Scientific, UK) was replaced. Images were taken immediately after the scratch was made, 24 h and 48 h using an Eclipse TE300 Inverted Phase Contrast Microscope (Nikon Instruments Incorporated, UK). Cell migration distance was calculated using Image J (1.47v National Institutes of Health, USA).

The inflammatory response to the calcium sulphate control cement (CS_Control) and SHRO loaded samples (CS_SHRO1 and CS_SHRO2) was determined using a human IL-8/CXCL8 enzyme-linked immunosorbent assay (ELISA) (R&D Systems, USA). Briefly, after 24 h of incubation 50 µL of cell culture media supernatant was taken from each well containing cells, media and cement samples (CS_Control, CS_SHRO1 and CS_SHRO2) and added to a well in the assay plate. Each well was diluted using 100 µL of assay diluent and incubated for 2 h at room temperature. Wells were then aspirated and washed before the addition of 100 µL of Human IL-8 conjugate. The assay plate was then incubated again for 1 h at room temperature before repeating the aspiration and washing step as before. 200 µL of assay substrate solution was then added before incubating further for 30 min. 50 µL of stop solution was then added as per manufacturer’s protocol. The optical density of each well was subsequently measured at 450 nm and was achieved using an Infinite F200 Pro (Tecan, UK).

### Statistical analysis

Statistical analysis was performed in GraphPad Prism V5.0 software. Two-way ANOVA was used to determine statistical differences between groups. A students t-test was used for post-hoc testing. An alpha value of 0.05 was used for all tests. Values of *p* < 0.05 were considered significant.

## Results

### Cement preparation

An overview of the preparation of each cement, as well as associated setting time, is provided in Table 1. Production of CS_Control, CS_SHRO1 and CS_SHRO2 cements with a L:P ratio of 0.2 mL/g resulted in workable pastes that could be transferred to a split mould and produce cylindrical test specimens. Initial and final setting times were determined using Gillmore needles. Calcium sulphate hemihydrate (CaSO_4_·0.5H_2_O) combined with water alone (CS_Control) resulted in the most rapidly setting paste, possessing an initial and final setting time of 10 and 20 min, respectively. Addition of SHRO as an initial component of the paste formulation (CS_SHRO1) extended the initial and final setting times to 30 and 40 min, respectively. In comparison, if the calcium sulphate hemihydrate (CaSO_4_·0.5H_2_O) cement was left to harden for 4 min prior to the addition of SHRO (CS_SHRO2), initial (20 min) and final (30 min) setting times were extended to a lesser extent than for CS_SHRO1.

**Table 1:** Overview of cement preparation and setting parameters. Setting times were acquired from n = 3 pastes and are recorded to the nearest 10 min.

Cement	Preparation overview	Preparation time	Initial setting time	Final setting time
CS_Control	Calcium sulphate hemihydrate and water, 30 s mixing	30 s	10 min	20 min
CS_SHRO1	Calcium sulphate hemihydrate, SHRO and water, 30 s mixing	30 s	30 min	40 min
CS_SHRO2	Calcium sulphate hemihydrate and water, 30 s mixing, wait 4 min, add SHRO, 30 s mixing	5 min	20 min	30 min

### Cement characterisation

**Figure 1:**
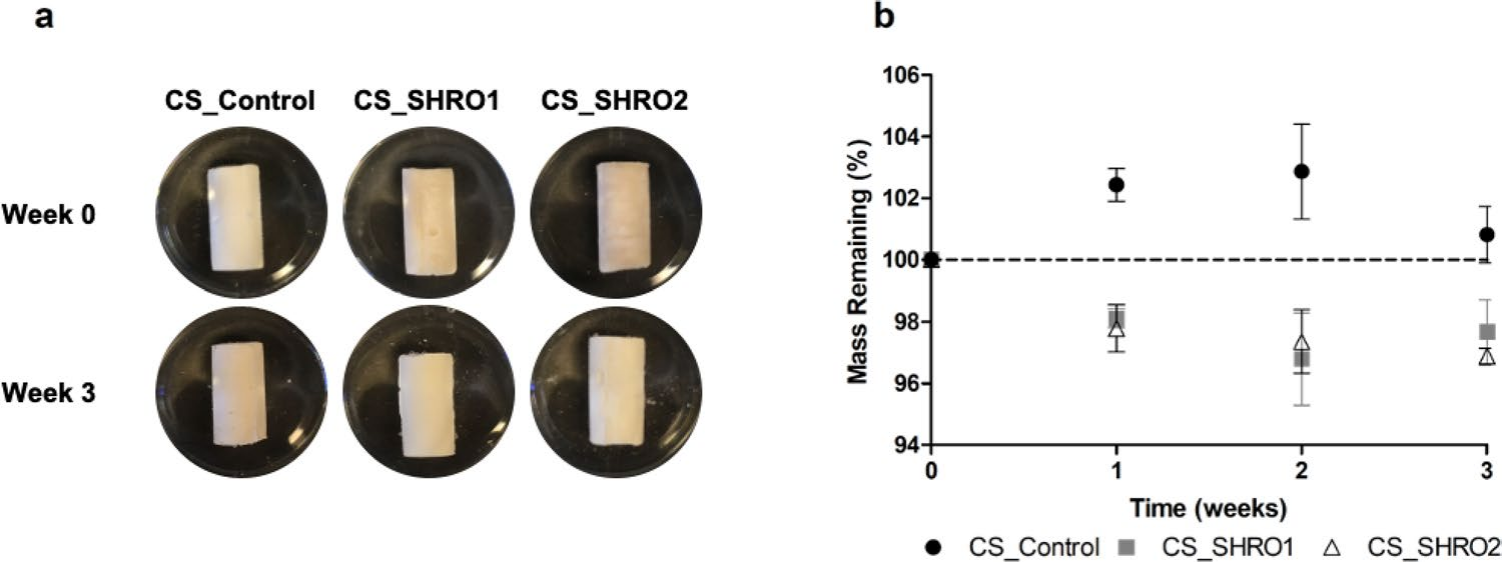
(**a**) Appearance and (**b**) measured changes in mass for CS_Control, CS_SHRO1 and CS_SHRO2 cements as prepared (Week 0) and following 3 weeks of static ageing in distilled water (Week 3) (mean ± SD, n = 3) (GraphPad Prism V5.0).

Cement degradation was examined by statically ageing specimens in distilled water over a period of 3 weeks. All specimens remained intact with negligible evidence of either fragmentation or degradation (Fig. 1a). Weekly measurements showed minimal variation in mass of over a three week period, with a maximum 3% increase at week 2. In contrast, CS_SHRO1 and CS_SHRO2 cements were both shown to lose 2–4% mass by week 3 and exhibited comparable degradation kinetics to each other (Fig. 1b).

**Figure 2:**
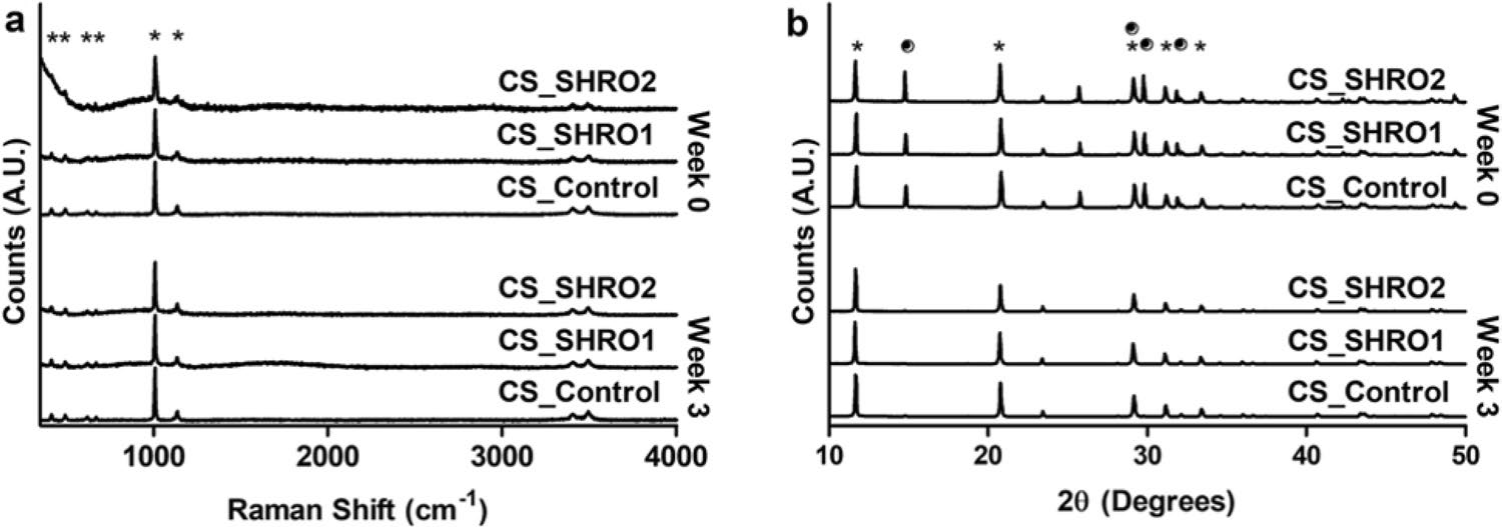
(**a**) Raman spectra and (**b**) XRD powder diffraction patterns for CS_Control, CS_SHRO1 and CS_SHRO2 cements as prepared (Week 0) and following 3 weeks of static ageing in distilled water (Week 3)**.** Peaks corresponding to CaSO_4_·0.5H_2_O are indicated with a (•) and those relating to CaSO_4_·2H_2_O are shown with a (*) (GraphPad Prism V5.0).

Raman spectroscopy confirmed the predominant phase of CS_Control, CS_SHRO1 and CS_SHRO2 cements as calcium sulphate dihydrate (CaSO_4_·2H_2_O) (Fig. 2a). Vibrational modes indicative of SO_4_ tetrahedra were identified in all samples. Consistent with calcium sulphate dihydrate (CaSO_4_·2H_2_O) peaks were identified at 1009 cm^-1^ (symmetric stretching, υ^1^), 415 cm^−1^ and 493 cm^−1^ (symmetric bending, υ^2^), 1137 cm^-1^ (antisymmetric stretching, υ^3^), and 620 cm^−1^ and 670 cm^−1^ (antisymmetric bending, υ^4^).

Prior to ageing XRD analysis demonstrated that all samples consisted of two crystalline phases (Fig. 2b). Diffraction peaks at 15, 29, 30 and 32° 2θ (•) were matched to the star rated ICDD pattern for CaSO_4_·0.5H_2_O (01-083-0438). The remaining peaks at 12, 21, 29, 31 and 33° 2θ (*) were matched to the star rated ICDD pattern for CaSO_4_·2H_2_O (01-070-0983). Quantification of phases revealed that CaSO_4_·0.5H_2_O and CaSO_4_.2H_2_O constitute all pre-aged cement samples at an approximate ratio of 1:2. Following ageing, only minor quantities (< 5%) of CaSO_4_·0.5H_2_O remained in any of the cement scaffolds due to further hydration within the aqueous environment (Table 2).

**Table 2:** Overview of cement composition as prepared (Week 0) and following 3 weeks of static ageing in distilled water (Week 3).

Cement	Week 0		Week 3	
	CaSO_4_·0.5H_2_O (%)	CaSO_4_·2H_2_O (%)	CaSO_4_·0.5H_2_O (%)	CaSO_4_·2H_2_O (%)
CS_Control	36.6	63.4	4.3	95.7
CS_SHRO1	34.7	65.3	2.2	97.8
CS_SHRO2	39.8	60.2	3.5	96.5

**Figure 3:**
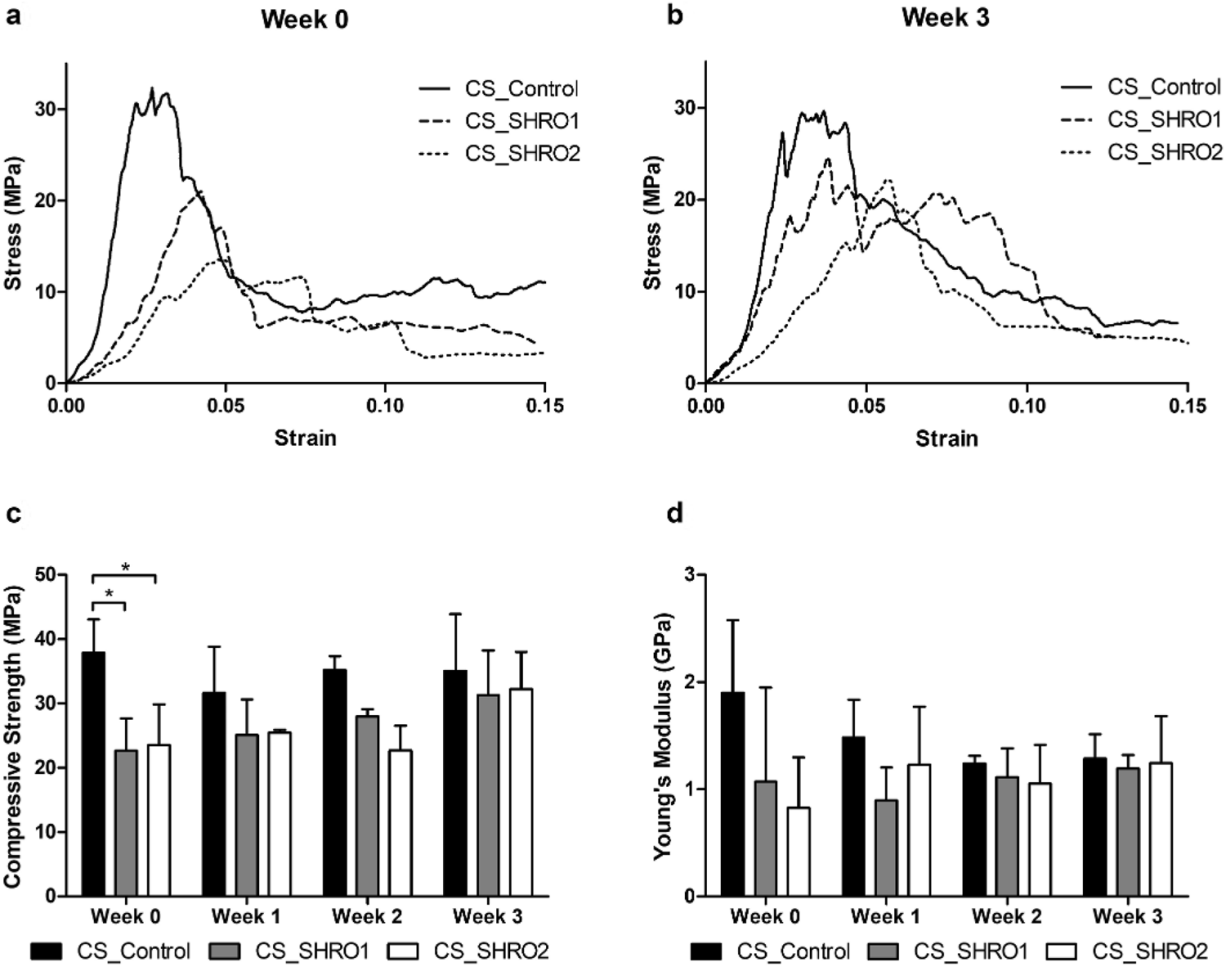
Average compressive stress vs. strain curves for CS_Control, CS_SHRO1 and CS_SHRO2 cements (**a**) as prepared (Week 0) and following (**b**) 3 weeks of static ageing in distilled water (n = 3). (**c**) Compressive strength and (**d**) Young’s modulus properties for CS_Control, CS_SHRO1 and CS_SHRO2 cements as prepared (Week 0), and following 1 week, 2 weeks and 3 weeks of static ageing in distilled water (Week 1, Week 2 and Week 3 respectively) (mean ± SD, n = 3) (GraphPad Prism V5.0).

The mechanical properties of the cement specimens were assessed using compression testing. Stress-stain curves revealed distinct regions of elastic and plastic deformation, reflective of the specimens catastrophically failing and then progressively fracturing in multiple locations (Fig. 3a,b). Average compressive strength and Young’s modulus were determined (Fig. 3c,d). Compressive strength values of 37.9 ± 5.2, 22.6 ± 5.0 and 23.5 ± 6.4 MPa were achieved by unaged CS_Control, CS_SHRO1 and CS_SHRO2 samples respectively with the unaged CS_Control demonstrating a significant (*p* < 0.05) increase in compressive strength in comparison to both CS_SHRO1 and CS_SHRO2 cements. Following 3 weeks of ageing, there was no significant difference in compressive strength between CS_Control, CS_SHRO1 and CS_SHRO2 samples (35.0 ± 8.9 MPa, 31.3 ± 7.0 MPa and 32.2 ± 5.8 MPa respectively). In terms of Young’s modulus, no significant differences were observed between CS_Control (1.9 ± 0.7 GPa), CS_SHRO1 (1.1 ± 0.9 GPa) and CS_SHRO2 (0.8 ± 0.5 GPa) specimens prior to ageing. Similarly, no significant differences were found after degradation experiments. Moreover, the compressive strength and Young’s modulus values of samples before and after ageing (3 weeks) were analysed using a t-test and all exhibited *p*-values of > 0.05.

**Figure 4:**
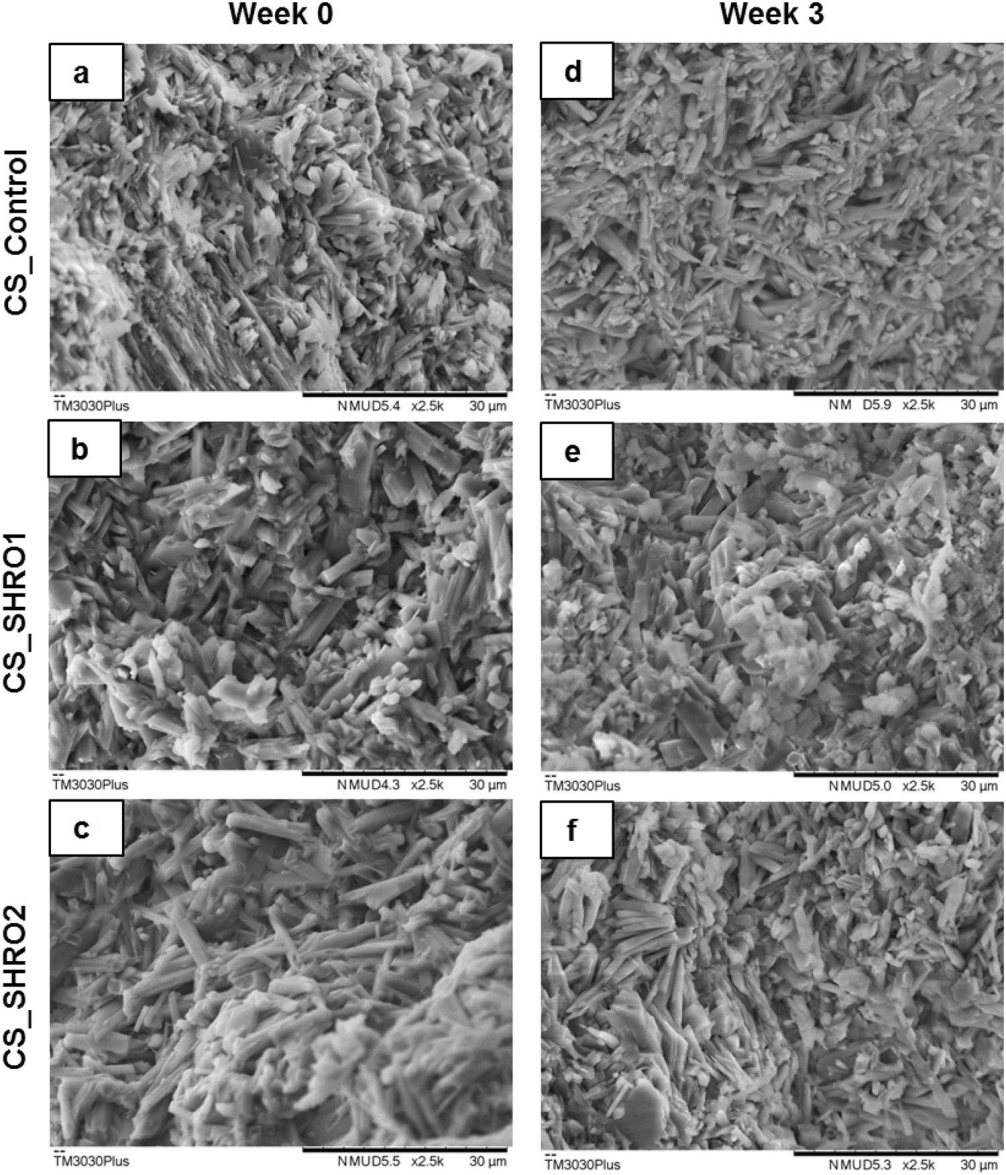
SEM images of (**a**) CS_Control, (**b**) CS_SHRO1 and (**c**) CS_SHRO2 cement fracture surfaces as prepared (Week 0), and (**d**) CS_Control, (**e**) CS_SHRO1 and (**f**) CS_SHRO2 cement fracture surfaces following 3 weeks of static ageing in distilled water (Week 3).

Cement fracture surfaces were analysed using SEM prior to and following ageing. As prepared CS_Control, CS_SHRO1 and CS_SHRO2 cements appeared to consist of a dense, irregular, network of interconnected high-aspect ratio crystals typical of both CaSO_4_·0.5H_2_O and CaSO_4_·2H_2_O (Fig. 4a–c). Individual crystal units exhibited approximate dimensions of 3 × 3 × 15 µm in all samples. Cements subjected to ageing showed no notable deviation in microstructure compared to as prepared cements and further between specimen groups (Fig. 4d–f).

### Antibiotic efficacy and cellular response

**Figure 5:**
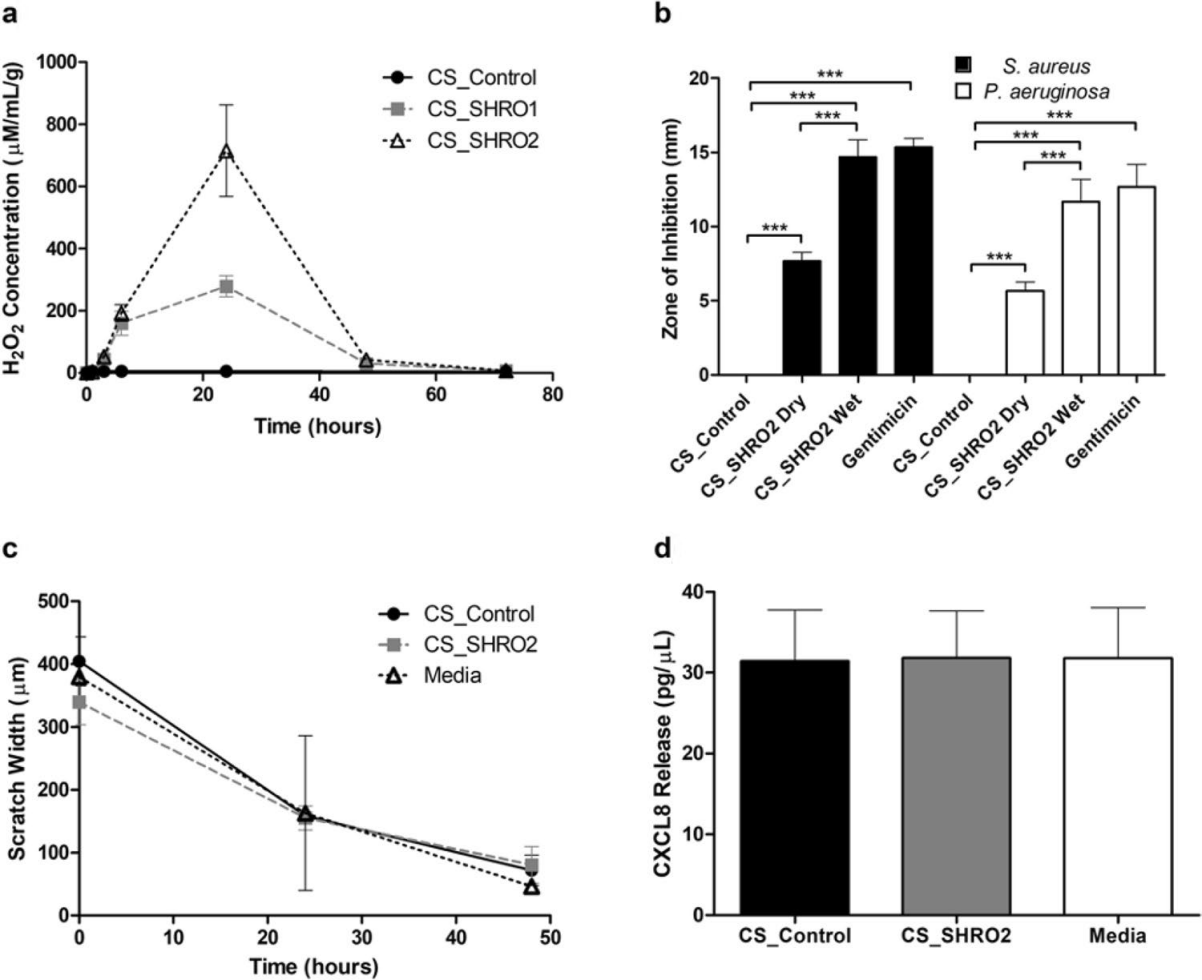
(**a**) Normalised H_2_O_2_ release for CS_Control, CS_SHRO1 and CS_SHRO2 cements in distilled water (mean ± SD, n = 3). (**b**) Zones of Inhibition for *Staphylococcus aureus* (*S. aureus*) and *Pseudomonas aeruginosa* (*P. aeruginosa*) in the presence of CS_Control, CS_SHRO1 and CS_SHRO2 cement supernatants (mean ± SD, n = 3). (**c**) Scratch assay and (d) human IL-8/CXCL8 enzyme-linked immunosorbent assay to determine the cellular effect of CS_Control and CS_SHRO2 scaffolds in comparison to media alone on wound healing using osteoblast cells (n = 2) (GraphPad Prism V5.0).

The generation of H_2_O_2_ from CS_Control, CS_SHRO1 and CS_SHRO2 cements in distilled water was measured over a period of 72 h (Fig. 5a). CS_Control cements consistently produced baseline levels of up to 5 μM/mL/g H_2_O_2_ while peak concentrations of 278.6 ± 34.1 and 715.0 ± 147.3 μM/mL/g were detected at 24 h for CS_SHRO1 and CS_SHRO2, respectively. After this time, H_2_O_2_ levels returned to baseline concentrations after 48 h.

Having demonstrated higher H_2_O_2_ peak values, CS_SHRO2 cements were tested for their antimicrobial potency in pre-set (dry) and wet states (Fig. 5b). Levels of H_2_O_2_ generated from all CS_SHRO2 samples were sufficient for inhibiting the growth of both *S. aureus* and *P. aeruginosa.* H_2_O_2_ enriched supernatant collected after 24 h from pre-set CS_SHRO2 cylinders gave zones of inhibition measuring 7.7 ± 0.6 mm for the Gram-positive *S. aureus*, whilst zones of inhibition for the Gram-negative *P. aeruginosa* measured at 5.7 ± 0.6 mm. This was significantly smaller (*p* < 0.05) than those measured for samples set in situ, which produced zones of inhibition measuring 14.7 ± 1.2 and 11.7 ± 1.0 mm for *S. aureus.* and *P. aeruginosa,* respectively. Promisingly, CS_SHRO2 set in situ showed no significant difference in inhibition to that of a comparable dose of gentamicin producing zones of 15.3 ± 0.6 (*S. aureus*) and 12.7 ± 1.5 mm (*P. aeruginosa)*.

A scratch assay was performed in the presence of CS_Control and CS_SHRO2 cements in order to determine the effect on osteoblast migration as a simple model for wound healing (Fig. 5c). The scratch (distance between cells) initially measured at 404.50 ± 38.9, 339.50 ± 36.1 and 380.00 ± 1.4 μm for CS_Control, CS_SHRO2 and media only samples, respectively. After 48 h it was shown that in all cases that cells migrated across the gap, reducing the initial size of the scratch by 82.2% (CS_Control), 76.29% (CS_SHRO2) and 87.76% (Media control). There was found to be no substantial difference in cell migration distance across the “wound gap” between samples and controls. Alongside the scratch assay, expression of inflammatory chemokines were assessed and no notable differences in CXCL8 level were observed between the CS_Control (31.40 ± 6.3 pg/μL), SHRO containing cements (31.83 ± 5.8 pg/μL) and media alone (31.78 ± 6.3 pg/μL) (Fig. 5d).

## Discussion

In recent decades the emergence of calcium-based scaffolds, such as calcium sulphate cement, to augment bone defects and provide a scaffold for cellular integration has proved highly successful. However, in order to enhance patient recovery these systems are often prophylactically loaded with antibiotics-commonly gentamicin. Antibiotics are a cornerstone of modern medicine and as such the current rapid evolution of resistant species is an alarming threat. This work has demonstrated a methodology to incorporate an alternative antimicrobial substance, specifically a reactive oxygen producing honey (SHRO), into a calcium sulphate cement. Importantly it is demonstrated that structural integrity of the SHRO containing cement is maintained while exhibiting comparable potency to an antibiotic control against Gram-negative and Gram-positive species.

The cement powder utilised in this work was an alpha type calcium sulphate hemihydrate (CaSO_4_·0.5H_2_O) that can results in a harder calcium sulphate dihydrate (CaSO_4_·2H_2_O) phase compared to beta type powders. Theoretically, less water is required for workable paste formation with alpha type plaster, which is ultimately crucial to the antimicrobial activity of the honey infused formulations following setting as discussed herein. It was observed that adding SHRO to calcium sulphate hemihydrate (CaSO_4_·0.5H_2_O) and water increased the time taken to form a completely hardened scaffold, which suggests that either the honey is altering the reaction products or the setting kinetics. The extent to which setting is delayed is dependent upon the timing of SHRO incorporation into the ceramic matrix. It is postulated that the osmotic nature of the SHRO actively removes the free water required for the dissolution of calcium sulphate hemihydrate (CaSO_4_·0.5H_2_O) and subsequently delays the precipitation of calcium sulphate dihydrate (CaSO_4_·2H_2_O) (Eq. 1). This is a theory supported by Raman spectroscopy and XRD, which indicates that no additional reaction products have been formed. Hall et al.^36^ demonstrated that the presence of additional water prior to application leads to premature ROS production and a reduction in antimicrobial efficacy at point of use. The osmotic nature of SHRO also allows for the SHRO to be incorporated into the developing ceramic matrix during preparation. SHRO draws water from the hardening matrix, acting as a liquefier, this allows for homogeneous mixing. In theory, less free water would be available for osmotic extraction by SHRO given the partial transformation into the stable hydrated calcium sulphate (CaSO_4_·2H_2_O) phase during the 4 min initial setting period.

It was hypothesised that locking water into the crystal structure of the hydrated calcium sulphate (CaSO_4_·2H_2_O) crystal structure would benefit the antimicrobial efficacy of CS_SHRO2 scaffolds, as this reduces the free water available to generate ROS species on contact with SHRO. Therefore, CS_SHRO2 cement will possess a greater potential to generate ROS following setting over CS_SHRO1 cement. Notably it was found that CS_SHRO2 released > 2.5 times more hydrogen peroxide (715.0 ± 147.3 μM/mL/g) over 24 h than CS_SHRO1 (278.6 ± 34.1 μM/mL/g) despite both formulations containing the same amount of active supporting the hypothesis that ROS production would be affected by free water availability. Currently, antibiotics are prescribed prophylactically in order to decrease postoperative infections, however antimicrobial prophylaxis is to be discontinued 24 h after surgery as stated by the guidelines^44^. The timeframe of antimicrobial activity for these cements therefore falls within the recommended guidelines. Interestingly however as the ROS falls rapidly after 24 h the concentrations of ROS could be beneficial for bone regeneration^19^.

Following preparation, there were notable differences in scaffold appearance between groups. Compared to CS_Control cements, which were pure white, SHRO infused CS_SHRO1 and CS_SHRO2 cements were distinguishable by a slight golden colouration. During ageing experiments, the supernatant surrounding CS_Control specimens remained clear. Within 24 h, the ageing supernatant of CS_SHRO1 and CS_SHRO2 specimens took on a golden hue. This is suggested to be an indicator of water infiltration within the internal structure of cements and the leeching of SHRO from these scaffolds. This may account for the mass reduction (2–4%) observed in the honey containing samples (Fig. 1b). As indicated by the Raman spectra and XRD patterns, calcium sulphate hemihydrate is detected in all of the prepared samples. Its presence is likely due to the cements being prepared with the least possible volume of water to form a workable paste, making water a limiting reagent of the setting reaction in order to restrict its interaction with SHRO and prevent premature production of ROS (Eq. 2). Unreacted calcium sulphate hemihydrate in the cement scaffolds was then found to convert to calcium sulphate dihydrate during the ageing experiment (Table 2), explaining why CS_Control cements gain 3% mass during the first 2 weeks of ageing. Following 3 weeks of ageing, the mass of CS_Control specimens was comparable to the week 0 cylinders, suggesting some degradation of the cement scaffold. Interestingly, the changes in mass are not monotonic, suggesting a dynamic relationship between mass increase due to the further precipitation of calcium sulphate dihydrate and the loss of scaffold due to the release of the therapeutic additive and cement degradation.

At week 0, SHRO addition was found to reduce the mechanical integrity of the materials as Control scaffolds (CS_Control) possessed higher compressive strength and Young’s modulus values compared to CS_SHRO1 and CS_SHRO2 (Fig. 3c–d). As the honey is not a particulate antimicrobial, it was not expected to act as a conventional stress riser within the bulk matrix^45^. Interestingly, it has previously been showed that the strength of calcium-based cement can be influenced by the addition of antibiotic compounds, possibly through mechanisms of chemical interaction during the setting period that can alter the scaffold microstructure^46^. Mechanical properties of cement scaffolds are highly dependent on microstructural features, such as pore size and pore distribution, which present weak points in the scaffold that may initiate crack formation^47,48^. Given that SHRO is osmotic and has a naturally low pH (3.8), it may influence the setting reaction and microstructural characteristics of calcium sulphate cement by drawing water away from the progressing reaction (Eq. 1). This could restrict precipitation and possibly promote pore formation. Although SHRO has the potential to influence the microstructure of cement, at the loading levels employed minimal deviations in microstructure were observed.

Increases in compressive strength and Young’s modulus from week 0 to week 3, though not significant between cement groups, may be the result of compositional evolution of the cements promoted by the ageing conditions. Calcium sulphate cements submerged in physiological media undergo hydration, absorbing water from the surrounding environment^49^. Consequently, the majority of remaining calcium sulphate hemihydrate (CaSO_4_·0.5H_2_O) comprising the cements at week 0 is transformed to the more stable calcium sulphate dihydrate (CaSO_4_·2H_2_O) by week 3 (Table 2), possibly accounting for increases in the physical attributes determined.

Ultimately, the resulting compressive strengths of the scaffolds exceed those of human trabecular bone harvested from various sites of the human body, including the mandible (0.2–11 MPa) and femoral head (3–8 MPa), the values are substantially lower than that of compact load bearing cortical bone (110 MPa)^50–52^. These scaffolds may therefore provide sufficient mechanical stability in augmentation sites within non-load bearing anatomical regions. Given that the scaffolds retain mechanical properties and undergo minimal degradation over the course of in vitro ageing, SHRO incorporated cements may be suitable as regenerative scaffolds over an extended period of time if required^53^.

It has been demonstrated that the release of ROS, such as hydrogen peroxide, from SHRO is pertinent to its antimicrobial efficacy^54^. CS_SHRO2 demonstrated greater capacity for the generation of hydrogen peroxide as per the hypothesis and as thus justifies the selection of CS_SHRO2 for further testing. CS_SHRO2 was tested for its antimicrobial efficacy against clinically relevant bacterial species^55^, by means of agar diffusion tests, examining the zones of inhibited growth on inoculated agar plates. The preparation of CS_SHRO2 cements for this in vitro assay was conducted in two ways, the first by forming pre-set cylinders that were then immersed in distilled water and incubated at 37 °C for 24 h. Hydrogen peroxide enriched supernatant was then removed and added to the wells in the agar. This methodology allows the antimicrobial efficacy of the cement post setting to be examined while also assessing whether this material could be used as a pre-set granule formulation to provide a prophylactic regenerative template. It was found that CS_SHRO2 pre-set cement does have the ability to inhibit the growth of both Gram-positive *S. aureus* and Gram-negative *P. aeruginosa*, however the zones of inhibition were significantly smaller (*p* < 0.05) than those of cements directly injected into the well and allowed to set (Fig. 5b). The reduced antibacterial potency of the pre-set formulation suggests that a degree of ROS activation occurs during hardening. To enable this novel antimicrobial cement to be used in its pre-set form it may be possible to increase the baseline efficacy of the incorporated SHRO, as reported by Dryden et al.^38^. Promisingly, when the CS_SHRO2 paste is administrated directly into the agar plate the resultant zones of inhibition were comparable to an equivalent dose of gentamicin. To enable a direct efficacy comparison between the ROS producing SHRO cement and gentamicin the same mass was used. The mass of gentamicin utilised is lower than what is currently commercially available, however both agents demonstrate the ability to kill Gram-positive and Gram-negative organisms, consistent with that of previous in vitro studies involving SHRO. As thus this further demonstrates the use of SHRO as a potential alternative to traditional antibiotics^14,36,38–40,54,56,57^. In addition, future work may also quantify efficacy.

Having assessed the potent antimicrobial viability of the CS_SHRO2 cement formulation it was necessary to investigate the effect these formulations would have upon endogenous osteoblast cells that would be present in the surrounding defect tissue. This was achieved by conducting a scratch assay and an ELISA detecting inflammatory chemokines. The scratch assay, a methodology described by Liang et al.^58^, is based upon observing and measuring the migration of the leading edge of a confluent cell monolayer, across an artificially formed gap “scratch”, until new cell to cell contact is achieved. No impairment of osteoblast migration was observed in the presence of CS_SHRO2 scaffolds, which is promising since maintenance of wound healing would be critical to patient recovery. This cellular compatibility was further supported by investigating the degree of inflammatory chemokines produced by the osteoblast cells. Interleukin 8 (CXCL8) is produced by cells, such as osteoblasts as an environmental immune response in order to stimulate macrophages recruitment to a particular site^59,60^. It was found that there was no notable difference in CXCL8 concentration in the media containing CS_Control cements supporting claims in the literature that calcium sulphate does not elicit an inflammatory response^61^. CS_SHRO2 scaffolds also exhibited this absence of an inflammatory response suggestive of a compatible relationship between the scaffold, released SHRO, and the endogenous cells. Further work however may be needed in more biologically relevant media to establish long term toxicity and whether the production and duration of the H_2_O_2_ is sufficient to prevent the development of late onset clinical infections. The later could be achieved by quantify the log reduction of bacterial load. In addition, alternatives to calcium sulphate cements could be explored. Calcium phosphate cements are frequently employed by clinicians for the augmentation of patient bone defects. Calcium phosphate cements composed of hydroxyapatite (Ca_10_(PO_4_)_6_(OH)_2_) or brushite (CaHPO_4_.2H_2_O) can be prepared from starting powders of differing calcium phosphate formulae mixed with a liquid solution component^1,62,63^. The inclusion of water can be utilised to produce not only reactive and hardening pastes, but also to promote the controlled generation ROS. As such, these systems may provide viable alternatives for SHRO modification of interest for further study.

## Conclusions

In conclusion this work addresses the need for the production of regenerative bone scaffolds that engender efficacious alternative antimicrobial solutions to use instead of traditional antibiotics. This has been achieved by formulating calcium sulphate cements that incorporate a reactive oxygen producing honey (SHRO). SHRO loaded calcium sulphate cement were found to be mechanically suitable for use in augmentation sites within non-load bearing anatomical regions.

The antimicrobial efficacy of SHRO is largely due to a water initiated and enzyme mediated oxidation of glucose, which produces the reactive oxygen species (ROS), which lead to microbial killing. By incorporating the SHRO into the developing scaffold matrix, it was hypothesised that the water would already be bound in the crystal structure of the hydrated calcium sulphate phase. This methodology of incorporation was shown to limit premature activation of ROS. In addition, in order for an antimicrobial effect to occur the local concentration of ROS must be > 25 µM, this efficacy was demonstrated after 24 h in vitro against clinically relevant Gram positive (*S. aureus*) and Gram negative (*P. aeruginosa*) bacteria. Furthermore, after 48 h this novel SHRO loaded calcium sulphate cement was not found to inhibit would healing or elicit an inflammatory response in osteoblast bone cells, supporting the theory that as ROS concentrations declines from that which proves an antimicrobial affect, concentration beneficial for wound healing (0.1–10 µM) may be generated. In summary, this study presents promising evidence that other antimicrobials, such as SHRO, may be used as alternatives to traditional antibiotics in bone cements, which is a timely development in the wake of the bacterial resistance crisis.
